# Retrospective analysis of systemic chemotherapy and total parenteral nutrition for the treatment of malignant small bowel obstruction

**DOI:** 10.1002/cam4.587

**Published:** 2015-12-29

**Authors:** Jay Chouhan, Rohan Gupta, Joe Ensor, Kanwal Raghav, David Fogelman, Robert A. Wolff, Michael Fisch, Michael J. Overman

**Affiliations:** ^1^ Department of Internal MedicineThe University of Texas Health Sciences CenterHoustonTexas; ^2^ Houston Methodist Cancer CenterHouston Methodist Research InstituteThe University of Texas MD Anderson Cancer CenterHoustonTexas; ^3^ Department of Gastrointestinal Medical OncologyThe University of Texas MD Anderson Cancer CenterHoustonTexas; ^4^ Department of General OncologyThe University of Texas MD Anderson Cancer CenterHoustonTexas

**Keywords:** Chemotherapy, gastrointestinal neoplasms, intestinal obstruction, ovarian neoplasms, survival analysis, total parenteral nutrition

## Abstract

Malignant small bowel obstruction (MSBO) that does not resolve with conservative measures frequently leaves few treatment options other than palliative care. This single‐institution retrospective study assesses the outcomes of a more aggressive approach—concurrent systemic chemotherapy and total parenteral nutrition (TPN)—in the treatment of MSBO. The MD Anderson pharmacy database was queried to identify patients who received concurrent systemic chemotherapy and TPN between 2005 and 2013. Only patients with MSBO secondary to peritoneal carcinomatosis requiring TPN for ≥8 days were included. Survival and multivariate analyses were performed using the Kaplan–Meier method and Cox proportional hazard models. The study included 82 patients. MSBO resolution was observed in 10 patients. Radiographic assessments showed a response to chemotherapy in 19 patients; 6 of these patients experienced MSBO resolution. Patients spent an average of 38% of their remaining lives hospitalized, and 28% of patients required admission to the intensive care unit. In multivariate modeling, radiographic response to chemotherapy correlated with MSBO resolution (odds ratio [OR] 6.81; 95% confidence interval [CI], 1.68–27.85, *P *= 0.007). Median overall survival (OS) was 3.1 months, and the 1‐year OS rate was 12.6%. Radiographic response to chemotherapy (HR 0.30; 95% CI, 0.16–0.56, *P *< 0.001), and initiation of new chemotherapy during TPN (HR 0.55; 95% CI, 0.33–0.94, *P *= 0.026) independently predicted for longer OS. Concurrent treatment with systemic chemotherapy and TPN for persistent MSBO results in low efficacy and a high morbidity and mortality, and thus should not represent a standard approach.

## Introduction

Malignant bowel obstruction (MBO) is a common complication of cancer; it occurs in 10–20% of colorectal cancer patients and 20–50% of ovarian cancer patients 1, 2, 3. Malignant small bowel obstruction (MSBO) poses the greatest challenge for clinical care. The outcomes for surgical intervention are generally poor, with several studies demonstrating a low likelihood of meaningful clinical benefit 4, 5, 6. In part, this relates to the frequent multifocality of small bowel involvement in the context of peritoneal carcinomatosis and the poor nutritional status of patients. Therefore, a conservative management approach consisting of bowel rest is considered standard treatment for symptomatic episodes of MSBO. It has been reported that 36% of cases with MBO achieve symptomatic relief with conservative treatment 7. However, those failing such strategies have goals of care that are typically palliative in nature and preclude the continuation or initiation of anti‐cancer therapy.

Supplemental total parenteral nutrition (TPN) has not been shown to improve outcomes in patients with terminal cancer 8. Outcomes from the use of supplemental TPN among patients with MBO are poor, with the median overall survival (OS) duration ranging from 53 to 140 days 9, 10, 11, 12, 13. Studies have reported even worse outcomes for patients with MBO in the context of peritoneal carcinomatosis, with median OS durations of 40–74 days with TPN 14, 15, 16. Though studies have frequently noted that a small subpopulation appears to benefit from TPN, recognizing members of this subgroup ahead of treatment has, thus far, proven difficult 16, 17, 18, 19.

The use of systemic chemotherapy in conjunction with TPN to manage MSBO is an aggressive approach that has not been well studied. Several small retrospective studies have included patients who have undergone this regimen 11, 17, 20, 21, 22, but no study has yet attempted to report on a large multitumor dataset. Because recent improvements in systemic chemotherapy have resulted in improved outcomes for patients with peritoneal carcinomatosis, a clearer understanding of the risks and benefits of aggressive TPN and chemotherapy treatment is needed 23, 24. In this study, we examine a large, single‐institution dataset to describe outcomes associated with concurrent TPN and systemic chemotherapy for persistent MSBO after conservative management.

## Methods

### Patient selection and data collection

We queried The University of Texas MD Anderson Cancer Center's pharmacy administration database to identify all patients treated concurrently with intravenous chemotherapy and TPN between January 2005 and December 2013 (*n* = 442). We excluded patients on the basis of nonsolid tumor histology (*n* = 219), age ≤16 years old (*n* = 12), administration of TPN for ≤8 days (*n* = 10), duodenal obstruction as cause of MSBO (*n* = 7), lack of radiographic or surgical confirmation of SBO (*n* = 111), and lack of peritoneal carcinomatosis (*n* = 1). The final study population consisted of 82 patients. The Institutional Review Board at MD Anderson Cancer Center approved this study.

Two physicians (J. C. and R. G.) retrieved from the database retrospective data on patient demographics, tumor characteristics, laboratory indices, chemotherapy and surgical treatments, TPN supplementation, frequency of hospitalization, and duration of stay in the inpatient setting. TPN‐related complications included in this study were line infections and hyperbilirubinemia. Line infection was defined as a positive blood or catheter‐tip culture from the patient's TPN line, and hyperbilirubinemia was defined as a total bilirubin level >1.5 times the upper limit of normal (1.0 mg/dL) that was unexplained by hepatic metastases, biliary obstruction, or prior hepatobiliary disease. Best response to chemotherapy was categorized semiquantitatively as stable disease, disease response, or disease progression based on a retrospective review of the patient's radiographic records and the treating physician's assessment of them. MSBO resolution was defined as a patient tolerating an oral diet without the need for additional intravenous nutritional supplementation for ≥60 days.

### Statistical analysis

Comparisons of relevant factors were made using analysis of variance (ANOVA), chi‐square tests, or log‐rank test. Kaplan–Meier curves were used to calculate time to event analyses, and were calculated from the initial date where TPN supplementation and chemotherapeutic intervention coincided until the date of last contact or occurrence of a relevant indicated event.

Multivariate Cox proportional hazard models were created and included any variable with a *P*‐value <0.10 in univariate analysis. Multivariate Cox proportional hazard modeling was conducting using a stepwise selection approach. A pool of candidate predictor variables consisting of ten covariates (age, body mass index, duration of metastatic disease, sex, site, histology, peritoneum only metastases, chemotherapy regimen, new chemotherapy start on TPN, and line of therapy) were offered to the model. Factors significant at the 0.10 level were allowed to enter the model, but significance at the 0.05 level was required to stay in the model. A *P*‐value of 0.05 was considered statistically significant. All analysis was performed using SAS software, version 9.4 (SAS Institute Inc., Cary, NC).

## Results

### Patient characteristics and clinical course

Among the 82 patients with MSBO secondary to peritoneal carcinomatosis who met the inclusion criteria (Table 1), the median age was 55 years. The most common primary tumor types were gastrointestinal (59.8%) and gynecologic (22.0%); extra‐peritoneal metastases were present in 48 (58.5%) patients. The median follow‐up time was 89.5 days (range, 4–2117 days).

**Table 1:** Patient demographic baseline characteristics (*N* = 82)

Variable	*N*	Percentage or range
Median age, years	55	17–85
Median initial body mass index (kg/m^2^)	23.9	14.3–38.0
Median initial albumin (g/dL)	2.8	1.6–4.4
Female sex	51	62.2
Caucasian race	53	64.6
Median duration of metastasis prior to TPN/chemotherapy, months	6.9	0–115
Extraperitoneal metastasis	48	58.5
Previous abdominal surgery	59	72
Tumor histology		
Carcinoma	71	86.6
Noncarcinoma	11	13.4
Primary site		
Gastrointestinal	49	59.8
Colorectal	20	
Appendix	6	
Pancreas	6	
Other^a^	11	
Gynecological	18	22.0
Ovarian/Primary Peritoneal	16	
Uterine	2	
Other^a^	15	18.3
Line of chemotherapy		
1st	38	46.3
2nd	15	18.3
≥3rd	29	35.3
First chemotherapy regimen		
5‐FU	32	39
Taxane	15	18.3
Other^b^	35	42.7
New chemotherapy start during TPN	58	70.7

TPN, total parenteral nutrition.

Stomach (5); gastrointestinal cancer of unknown primary (1); gallbladder (1); small bowel carcinoid (2); small bowel adenocarcinoma (1); and intra‐abdominal desmoid tumor (1).

a Sarcoma (4); melanoma (4); cancer of unknown primary (3); bladder (2); urachus; (1); and prostate (1).

b 5‐FU + taxane (5); doxorubicin (6); gemcitabine (2); irinotecan (5); platinum‐based therapy (7); carmustine (1); anti‐EGFR therapy (2); pemetrexed (1); IL‐2 (2); topotecan (2); and bevacizumab.

The median duration of metastatic disease prior to the administration of concurrent systemic chemotherapy and TPN was 6.9 months (range, 0–115 months). The initial systemic chemotherapy was the first‐line therapy for metastatic disease in 38 (46.3%) patients. Twenty‐four (29.3%) patients continued to receive the same chemotherapy regimen after the initiation of TPN. The median duration of chemotherapy prior to TPN initiation was 8.5 days (range, 1–87 days), with only one patient having more than 2 cycles of chemotherapy prior to TPN initiation.

Response to chemotherapy was assessed radiographically in 54 (65.9%) patients (Table 2). The remaining 28 (34.1%) patients demonstrated clinical decline and did not undergo further radiographic imaging. Of the 54 patients with radiographic assessments, 19 patients showed a radiographic response, 9 had stable disease, and 26 had progressive disease (Table 2).

**Table 2:** Treatment outcomes (*N* = 82)

Variable	*N*	Percentage or range
Small bowel resolution^a^^,^ b	10	12.2
Median overall survival, months	3.1	0.03–69.4
Chemotherapy outcome		
Best radiographic response		
Response	19	23.2
Stable	9	11.0
Progression	26	31.7
Unknown	28	34.1
Treatment course		
Median duration of TPN, days	45	(9–639)
Subsequent hospitalization	63	76.8
Median number of hospitalizations	2	1–11
Median inpatient stay, days	26.5	4–167
Intensive care unit stay	23	28.1
TPN‐related complications	27	32.9
Line infection	17	
Hyperbilirubinemia	10	
Bowel perforation	4	4.9
Venting gastrostomy tube insertion	56	68.3

TPN, total parenteral nutrition.

a Defined as an oral diet without intravenous nutritional supplementation for ≥60 days.

b Tumor types were ovarian (4); prostate (1); small bowel adenocarcinoma (1); appendiceal adenocarcinoma (1); small bowel carcinoid (1); endometrioid carcinoma (1); and abdominal desmoid (1).

The median duration of TPN was 45 days (range, 9–639). TPN‐related complications (i.e., line infections or hyperbilirubinemia) occurred in 27 (32.9%) patients. Bowel perforation occurred rarely, in only 4 (4.9%) patients. Sixty‐three (76.8%) patients required hospitalization after the initiation of systemic chemotherapy and TPN, with 23 (28.1%) patients admitted to the intensive care unit. The median number of hospitalizations was 2 (range, 1–11), and the median time spent hospitalized was 26.5 days (range, 4–167 days). On average, patients spent 38% (range, 1–100%) of their remaining lives in the hospital.

### Resolution of MSBO

**Figure 1:**
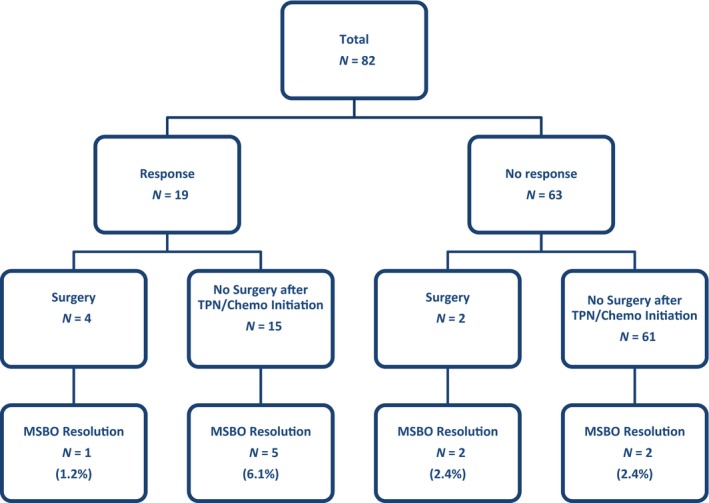
Malignant small bowel obstruction (MSBO) resolution stratified by tumor response and treatment variables. TPN, total parenteral nutrition.

MSBO resolution was observed in 10 (12.2%) patients (Table 2; Fig. 1). As shown in Figure 1, resolution of MSBO was attributable to surgery in 3 cases and chemotherapy in 5 cases, while an additional two cases had MSBO resolution without either surgery or a chemotherapy response. Recurrence of the MSBO obstruction occurred in 6 of the 10 patients, at a median time of 5.3 months (range, 2–90 months) after the initial resolution. Surgery with the goal of correcting the MSBO was attempted infrequently (6 cases, 7.3%) with the median time from initiation of systemic chemotherapy and TPN to MSBO surgery of 35.5 days (range, 6–217 days).

On univariate analysis, surgery (odds ratio [OR] 9.86; 95% confidence interval [CI], 1.66–58.40, *P *= 0.01) and radiographic response to treatment (OR 6.37; 95% CI, 1.63–24.85, *P *= 0.007) were found to be predictors of MSBO resolution (Table 3). On multivariate modeling that did not include subsequent treatment variables of radiographic response or surgery for MSBO—no factors were found to be significant predictors of MSBO resolution. With the inclusion of the two subsequent‐treatment factors, only radiographic response correlated with an increased rate of MSBO resolution (OR 6.81; 95% CI, 1.68–27.63, *P *= 0.007).

**Table 3:** Univariate and multivariate analyses of factors correlating with resolution of malignant small bowel obstruction

		Univariate		Multivariate	
Variable	*n*	Odds ratio (95% CI)	*P*‐value^a^	Odds ratio (95% CI)	*P*‐value^a^
Age^b^	82	0.96 (0.91–1.00)	0.06		
Albumin^b^	79	1.06 (0.42–2.71)	0.90		
Body mass index^b^	81	0.98 (0.88–1.09)	0.68		
Metastatic disease duration ≥7 months	82	0.41 (0.10–1.69)	0.22		
New chemotherapy start during TPN	58	1.52 (0.33–6.94)	0.59		
Sex					
Male	31	Reference			
Female	51	0.87 (0.24–3.23)	0.84		
Site					
Gastrointestinal	49	Reference			
Gynecological	18	1.23 (0.24–6.28)	0.81		
Other	15	2.27 (0.50–10.32)	0.29		
Histology					
Carcinoma	71	Reference			
Noncarcinoma	11	4.78 (1.00–22.87)	0.051		
Extraperitoneal metastasis					
Yes	34	Reference			
No	48	0.97 (0.26–3.56)	1.00		
Chemotherapy regimen					
5‐FU	32	Reference			
Taxane	15	1.17 (0.21–4.50)	0.86		
Other	35	1.08 (0.26–4.50)	0.92		
Line of chemotherapy					
1st	38	Reference			
2nd	15	0.93 (0.18–4.75)	0.93		
≥3rd	29	1.09 (0.21–5.69)	0.92		
Surgery for MSBO					
No	76	Reference			
Yes	6	9.86 (1.66–58.40)	0.01		
Radiographic response					
No	63	Reference		Reference	
Yes	19	6.37 (1.63–24.85)	0.007	6.81 (1.68–27.63)	0.007

CI, confidence interval; TPN, total parenteral nutrition; MSBO, malignant small bowel obstruction.

a *P* < 0.05 was considered statistically significant.

b Continuous variable used to calculate odds ratios.

### Overall survival

**Figure 2:**
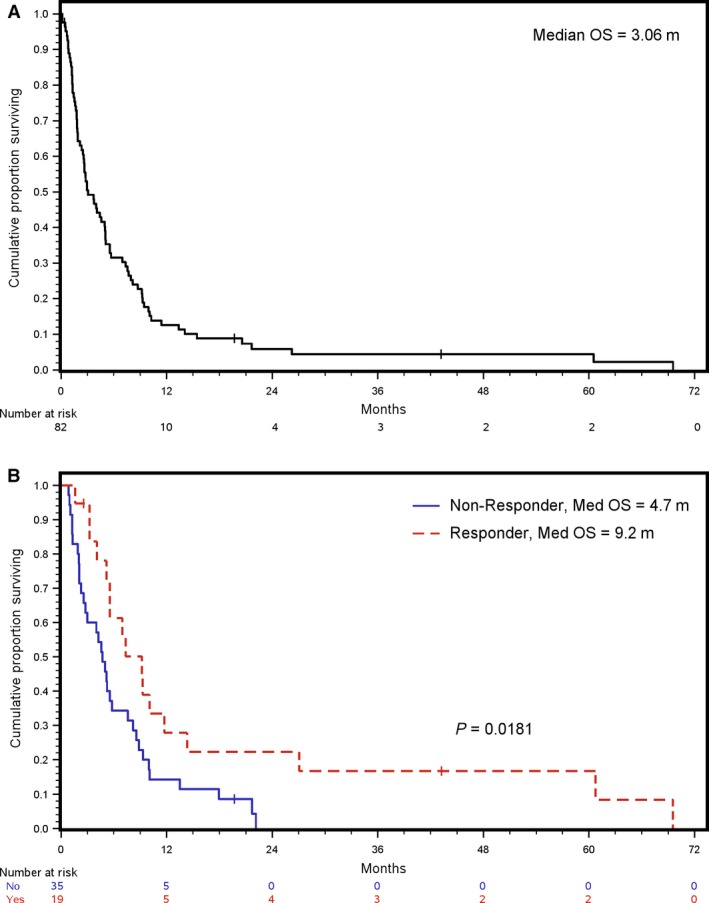
Overall survival (OS) for (A) all patients (*n* = 82) and (B) patients who underwent radiographic tumor assessment (*n* = 54).

The median OS duration for our patient cohort was 3.1 months (range, 0.03–69.4 months), with a 1‐year OS rate of 12.2% (Fig. 2A). Among the 54 patients who underwent radiographic assessments, those with a radiographic response to therapy showed longer OS than those with no response (median OS 9.2 vs. 4.7 months, *P *= 0.018) (Fig. 2B). The median OS was 1.7 months for patients who did not undergo radiographic evaluation. Of the 10 patients who lived longer than 1 year, three had favorable tumor types (desmoid, appendiceal adenocarcinoma and carcinoid) while the remaining 7 had ovarian/primary peritoneal (*n* = 4), prostate (*n* = 1), endometrial adenocarcinoma (*n* = 1), and small bowel adenocarcinoma (*n* = 1). The median time spent inpatient was 49 days (range 7–126 days) and SBO resolution occurred in 5 patients (2 following surgery and 3 following chemotherapy). Of the seven patients with nonfavorable tumor types, four underwent frontline chemotherapy and all had either a response or stable disease to chemotherapy.

On univariate analysis, only radiographic response to chemotherapy showed a statistically significant correlation with OS (OR 0.36; 95% CI, 0.20–0.65, *P *= 0.0007) (Table 4). Multivariate modeling that excluded the subsequent‐treatment variables showed no statistically significant factors. With the inclusion of radiographic response and MSBO surgery in the multivariate analysis, radiographic response (OR 0.30; 95%, 0.16–0.56, *P *= 0.004) and initiation of new systemic chemotherapy during TPN (OR 0.55; 95% CI, 0.33–0.94, *P *= 0.026) were correlated with improved OS.

**Table 4:** Univariate and multivariate analyses of factors correlating with overall survival

		Univariate		Multivariate	
Variable	*n*	Hazard ratio (95% CI)	*P*‐value^a^	Hazard ratio (95% CI)	*P*‐value^a^
Age^b^	82	1.00 (0.98–1.01)	0.70		
Albumin^b^	79	0.94 (0.68–1.30)	0.71		
Body mass index^b^	81	1.01 (0.97–1.04)	0.68		
Metastatic disease duration ≥7 months	82	0.99 (0.98–1.00)	0.94		
New chemotherapy start during TPN	58	0.77 (0.47–1.28)	0.3	0.55 (0.33–0.94)	0.026
Sex					
Male	31	Reference			
Female	51	0.90 (0.56–1.44)	0.67		
Site					
Gastrointestinal	49	Reference			
Gynecological	18	0.70 (0.49–1.27)	0.20		
Other	15	1.01 (0.56–1.83)	0.97		
Histology					
Carcinoma	71	Reference			
Noncarcinoma	11	0.93 (0.56–1.83)	0.84		
Extraperitoneal metastasis					
Yes	34	Reference			
No	48	0.92 (0.58–1.44)	0.70		
Chemotherapy regimen					
5‐FU	32	Reference			
Taxane	15	0.58 (0.30–1.14)	0.16		
Other	35	1.09 (0.65–1.84)	0.74		
Line of chemotherapy					
1st	38	Reference			
2nd	15	1.19 (0.64–2.21)	0.58		
≥3rd	29	1.38 (0.72–2.62)	0.33		
Surgery for MSBO					
No	76	Reference			
Yes	6	0.67 (0.32–1.39)	0.28		
Radiographic response					
No	63	Reference		Reference	
Yes	19	0.36 (0.20–0.65)	0.0007	0.30 (0.16–0.56)	0.0004

CI, confidence interval; TPN, total parenteral nutrition; MSBO, malignant small bowel obstruction.

a *P* < 0.05 was considered statistically significant.

b Continuous variable used to calculate odds ratios.

## Discussion

The treatment approach for cancer patients with MSBO after conservative management with bowel rest is poorly defined. Studies have shown limited benefit with either surgical intervention or supplemental TPN for this patient population 4, 5, 6, 9, 14, 16, 20. Our study analyzed the largest available dataset evaluating the more aggressive approach of concurrent systemic chemotherapy and TPN. We conclude that the outcomes of this aggressive approach are poor, yielding a median OS duration of 3.1 months. In addition, we observed significant treatment‐related morbidity; 76.8% of patients were rehospitalized, and on average they spent approximately 38% of their remaining lives in the hospital. Although a radiographic response to chemotherapy predicted MSBO resolution in our multivariate analysis, only 5 of the 19 patients with a radiographic response (6% of the entire cohort) experienced MSBO resolution attributable to the use of systemic chemotherapy. These results demonstrate that the aggressive treatment of MSBO secondary to peritoneal carcinomatosis with systemic chemotherapy and concurrent TPN supplementation provided minimal benefit to our patient population.

Several previous studies, with populations ranging from 4 to 31 patients, have reported on the palliative management of MBO with chemotherapy and TPN supplementation 11, 17, 18, 19, 20, 22. Brard et al. 10 conducted one of the larger studies to specifically address the role of TPN and chemotherapy in the setting of MBO; they found no statistically significant difference between the median OS duration of 18 patients treated with chemotherapy and TPN compared and that of 7 patients treated with chemotherapy alone (72 vs. 42 days, *P *= 0.09) 10. In contrast, Abu‐Rustum et al. 23 reported, in a study of 21 ovarian cancer patients with MBO, that patients receiving TPN and chemotherapy had a significantly longer median OS than those treated with chemotherapy alone (80 vs. 62 days, *P *= 0.031) 21. However, due to the retrospective nature of these studies and their relatively small sample sizes, no definitive conclusions can be drawn from them.

The frequency of MSBO resolution in our overall cohort (12.2%) was similar to that observed in two previous studies, which found MBO resolution in 1 (12.5%) of 8 and 2 (12.2%) of 11 ovarian cancer patients managed with an aggressive approach like the one examined in our study 20, 21. Among our patient cohort, radiographic response was the only independent predictor of MSBO resolution. However, a discrepancy between treatment response and MSBO resolution did exist. Of 19 patients with radiographic tumor response, only 5 (26%) patients went on to have MSBO resolution that could be attributed to chemotherapeutic intervention. A relationship between chemotherapy response and MBO‐resolution has not been well described in the literature, but was suggested in an examination of 39 ovarian cancer patients palliatively managed with chemotherapy and/or surgery. In this study, a platinum response disease was seen in 46% of patients with resolution of their MBO compared to only 27% of patients without resolution of their MBO, *P* = 0.17 25.

In addition, we identified no baseline factors that predicted either OS or MSBO resolution. Indeed, we were surprised that neither the line of chemotherapy used nor the tumor type predicted either outcome. It was also of interest that worse OS was seen in patients in whom the same chemotherapy administered prior to the development of MSBO was continued unchanged after the MSBO diagnosis. Given that treatment response was the only factor that correlated with both OS and MSBO resolution in our study, it is critical that, if this aggressive approach is attempted, the radiographic results from systemic chemotherapy are incorporated into clinical assessments of the risks and benefits of further treatment. However, the absence of any pretreatment factors that statistically correlated with improved outcomes poses a major limitation to the clinical application of this aggressive approach.

Though we were unable to assess quality of life measures due to our study's retrospective nature, our analyses suggest that the aggressive treatment approach is associated with high morbidity rates. In particular, 23 (28.1%) patients required admission to the intensive care unit, and 27 (32.9%) developed TPN‐related complications. In part, these findings may reflect the well‐known increased risks of infections and iatrogenic complications from the concurrent use of supplemental TPN in patients treated with chemotherapy 8. Most importantly in terms of quality of life, patients spent an average of 38% of their remaining lives in the hospital.

Although we examined the largest population of patients with MSBO treated with concurrent chemotherapy and TPN of any study to date, our study, like others, was limited by its relatively small cohort size and its retrospective nature. In addition, while our study population was representative of clinical practice patterns for the treatment of solid tumors, heterogeneity in the patient population and in tumor types prevented us from undertaking a more detailed investigation of the prognostic significance of various subgroups. Additionally, while better performance status has previously been reported to predict longer OS in patients with MBO 12, 16, 26, we were unable to evaluate this relationship due to a lack of documentation of performance status for hospitalized patients. Because the aggressive treatment approach may have only been considered for more functionally robust patients, it is possible that the population we studied reflects a highly selected cohort. A final limitation involves the definition of unresolving MSBO. By requiring more than 8 days of TPN, we designed our study criteria to identify patients with an unresolving MSBO that was likely not amenable to surgical intervention; nevertheless, a small subset of patients went on to have palliative surgical procedures, and 2 patients were observed to have spontaneous resolution of their MSBOs.

In conclusion we recommend against that the use of systemic chemotherapy and TPN for patients with MSBO secondary to peritoneal carcinomatosis. Though a small subset of the studied patients did gain a modest benefit, the lack of predefined factors that could identify this population in advance makes it clinically challenging to apply this aggressive approach, especially in light of the significant treatment‐related morbidity we observed.

## Conflict of Interest

None declared.

## References

[1] Ripamonti, C. , and E. Bruera . 2002 Palliative management of malignant bowel obstruction. Int. J. Gynecol. Cancer 12: 135–143. 11975672 10.1046/j.1525-1438.2002.01103.x

[2] Feuer, D. J. , K. E. Broadley , J. H. Shepherd , and D. P. Barton . 2000 Surgery for the resolution of symptoms in malignant bowel obstruction in advanced gynaecological and gastrointestinal cancer. Cochrane Database Syst. Rev. CD002764. 11034757 10.1002/14651858.CD002764

[3] Feuer, D. J. , K. E. Broadley , J. H. Shepherd , and D. P. Barton . 1999 Systematic review of surgery in malignant bowel obstruction in advanced gynecological and gastrointestinal cancer. The systematic review steering committee. Gynecol. Oncol. 75: 313–322. 10600282 10.1006/gyno.1999.5594

[4] Larson, J. E. , E. S. Podczaski , A. Manetta , C. W. Whitney , and R. Mortel . 1989 Bowel obstruction in patients with ovarian carcinoma: analysis of prognostic factors. Gynecol. Oncol. 35: 61–65. 2477316 10.1016/0090-8258(89)90012-7

[5] Turnbull, A. D. , J. Guerra , and H. F. Starnes . 1989 Results of surgery for obstructing carcinomatosis of gastrointestinal, pancreatic, or biliary origin. J. Clin. Oncol. 7: 381–386. 2918333 10.1200/JCO.1989.7.3.381

[6] van Ooijen, B. , van der Burg M. E. , A. S. Planting , P. D. Siersema , and T. Wiggers . 1993 Surgical treatment or gastric drainage only for intestinal obstruction in patients with carcinoma of the ovary or peritoneal carcinomatosis of other origin. Surg. Gynecol. Obstet. 176: 469–474. 8480270

[7] Tuca, A. , E. Guell , E. Martinez‐Losada , and N. Codorniu . 2012 Malignant bowel obstruction in advanced cancer patients: epidemiology, management, and factors influencing spontaneous resolution. Cancer Manag. Res. 4: 159–169. 22904637 10.2147/CMAR.S29297 PMC3421464

[8] 1989 Parenteral nutrition in patients receiving cancer chemotherapy. American college of physicians. Ann. Intern. Med. 110: 734–736. 2494922 10.7326/0003-4819-110-9-734

[9] August, D. A. , D. Thorn , R. L. Fisher , and C. M. Welchek . 1991 Home parenteral nutrition for patients with inoperable malignant bowel obstruction. JPEN J. Parenter Enteral. Nutr. 15:323–327. 1907683 10.1177/0148607191015003323

[10] Brard, L. , S. Weitzen , S. L. Strubel‐Lagan , N. Swamy , M. E. Gordinier , R. G. Moore , et al. 2006 The effect of total parenteral nutrition on the survival of terminally ill ovarian cancer patients. Gynecol. Oncol. 103: 176–180. 16564074 10.1016/j.ygyno.2006.02.013

[11] Chakraborty, A. , D. Selby , K. Gardiner , J. Myers , V. Moravan , and F. Wright . 2011 Malignant bowel obstruction: natural history of a heterogeneous patient population followed prospectively over two years. J. Pain Symptom Manage. 41: 412–420. 21131167 10.1016/j.jpainsymman.2010.05.007

[12] Cozzaglio, L. , F. Balzola , F. Cosentino , M. DeCicco , P. Fellagara , G. Gaggiotti , et al. 1997 Outcome of cancer patients receiving home parenteral nutrition. Italian society of parenteral and enteral nutrition (S.I.N.P.E.). JPEN J. Parenter. Enteral Nutr. 21: 339–342. 9406131 10.1177/0148607197021006339

[13] Chermesh, I. , T. Mashiach , A. Amit , N. Haim , I. Papier , F. Efergan , et al. 2011 Home parenteral nutrition (HTPN) for incurable patients with cancer with gastrointestinal obstruction: do the benefits outweigh the risks? Med. Oncol. 28: 83–88. 20107935 10.1007/s12032-010-9426-2

[14] Chen, C. J. , S. C. Shih , H. Y. Wang , F. J. Sun , S. C. Lu , C. H. Chu , et al. 2013 Clinical application of total parenteral nutrition in patients with peritoneal carcinomatosis. Eur. J. Cancer Care (Engl.) 22: 468–473. 23730735 10.1111/ecc.12052

[15] Pasanisi, F. , A. Orban , L. Scalfi , L. Alfonsi , L. Santarpia , E. Zurlo , et al. 2001 Predictors of survival in terminal‐cancer patients with irreversible bowel obstruction receiving home parenteral nutrition. Nutrition 17: 581–584. 11448576 10.1016/s0899-9007(01)00579-2

[16] Santarpia, L. , L. Alfonsi , F. Pasanisi , C. De Caprio , L. Scalfi , and F. Contaldo . 2006 Predictive factors of survival in patients with peritoneal carcinomatosis on home parenteral nutrition. Nutrition 22: 355–360. 16413750 10.1016/j.nut.2005.06.011

[17] Diver, E. , O. O'Connor , L. Garrett , D. Boruta , A. Goodman , M. Del Carmen , et al. 2013 Modest benefit of total parenteral nutrition and chemotherapy after venting gastrostomy tube placement. Gynecol. Oncol. 129: 332–335. 23402902 10.1016/j.ygyno.2013.02.002

[18] Duerksen, D. R. , E. Ting , P. Thomson , K. McCurdy , J. Linscer , S. Larsen‐Celhar , E. Brennenstuhl . 2004 Is there a role for TPN in terminally ill patients with bowel obstruction? Nutrition 20: 760–763. 15325683 10.1016/j.nut.2004.05.010

[19] King, L. A. , L. F. Carson , N. Konstantinides , M. S. House , L. L. Adcock , K. A. Prem , et al. 1993 Outcome assessment of home parenteral nutrition in patients with gynecologic malignancies: what have we learned in a decade of experience? Gynecol. Oncol. 51: 377–382. 8112649 10.1006/gyno.1993.1307

[20] Pothuri, B. , M. Montemarano , M. Gerardi , M. Gerardi , M. Shike , L. Ben‐Porat , P. Sabbaini , R. R. Barakat . 2005 Percutaneous endoscopic gastrostomy tube placement in patients with malignant bowel obstruction due to ovarian carcinoma. Gynecol. Oncol. 96: 330–334. 15661217 10.1016/j.ygyno.2004.09.058

[21] Abu‐Rustum, N. R. , R. R. Barakat , E. Venkatraman , and D. Spriggs . 1997 Chemotherapy and total parenteral nutrition for advanced ovarian cancer with bowel obstruction. Gynecol. Oncol. 64: 493–495. 9062158 10.1006/gyno.1996.4605

[22] Tunca, J. C. 1981 Impact of cisplatin multiagent chemotherapy and total parenteral hyperalimentation on bowel obstruction caused by ovarian cancer. Gynecol. Oncol. 12:219–221. 6795096 10.1016/0090-8258(81)90150-5

[23] Franko, J. , Q. Shi , C. D. Goldman , B. A. Pockaj , G. D. Nelson , R. M. Goldberg , et al. 2012 Treatment of colorectal peritoneal carcinomatosis with systemic chemotherapy: a pooled analysis of north central cancer treatment group phase III trials N9741 and N9841. J. Clin. Oncol. 30: 263–267. 22162570 10.1200/JCO.2011.37.1039 PMC3269953

[24] Grothey, A. , D. Sargent , R. M. Goldberg , and H. J. Schmoll . 2004 Survival of patients with advanced colorectal cancer improves with the availability of fluorouracil‐leucovorin, irinotecan, and oxaliplatin in the course of treatment. J. Clin. Oncol. 22: 1209–1214. 15051767 10.1200/JCO.2004.11.037

[25] Bryan, D. N. , R. Radbod , and J. S. Berek . 2006 An analysis of surgical versus chemotherapeutic intervention for the management of intestinal obstruction in advanced ovarian cancer. Int. J. Gynecol. Cancer 16: 125–134. 16445622 10.1111/j.1525-1438.2006.00283.x

[26] Bozzetti, F. , L. Cozzaglio , E. Biganzoli , G. Chiavernna, M. De Cicco, D. Donati, et al. 2002 Quality of life and length of survival in advanced cancer patients on home parenteral nutrition. Clin. Nutr. 21: 281–288. 12135587 10.1054/clnu.2002.0560

